# Comparative Epidemiological Assessment of Monkeypox Infections on a Global and Continental Scale Using Logistic and Gompertz Mathematical Models

**DOI:** 10.3390/vaccines11121765

**Published:** 2023-11-27

**Authors:** Obert Marín-Sánchez, Pedro Pesantes-Grados, Luis Pérez-Timaná, Olegario Marín-Machuca, Christian J. Sánchez-Llatas, Ruy D. Chacón

**Affiliations:** 1 Departamento Académico de Microbiología Médica, Facultad de Medicina, Universidad Nacional Mayor de San Marcos, Av. Carlos Germán Amezaga 375, Lima 15081, Peru; omarins@unmsm.edu.pe; 2 Unidad de Posgrado, Facultad de Ciencias Matemáticas, Universidad Nacional Mayor de San Marcos, Av. Carlos Germán Amezaga 375, Lima 15081, Peru; pedro.pesantes@unmsm.edu.pe; 3 Escuela Profesional de Genética y Biotecnología, Facultad de Ciencias Biológicas, Universidad Nacional Mayor de San Marcos, Av. Carlos Germán Amezaga 375, Lima 15081, Peru; luis.perez30@unmsm.edu.pe; 4 Departamento Académico de Ciencias Alimentarias, Facultad de Oceanografía, Pesquería, Ciencias Alimentarias y Acuicultura, Universidad Nacional Federico Villarreal, Calle Roma 350, Miraflores 15074, Peru; omarin@unfv.edu.pe; 5 Department of Genetics, Physiology, and Microbiology, School of Biology, Complutense University of Madrid (U.C.M.), C. de José Antonio Nováis, 12, 28040 Madrid, Spain; chrsan01@ucm.es; 6 Department of Pathology, School of Veterinary Medicine, University of São Paulo, Av. Prof. Orlando M. Paiva, 87, São Paulo 05508-270, Brazil

**Keywords:** basic reproduction number, cases, coefficient of determination, critical time, Gompertz function, logistic regression, mathematical modeling, monkeypox

## Abstract

The monkeypox virus (MPXV) has caused an unusual epidemiological scenario—an epidemic within a pandemic (COVID-19). Despite the inherent evolutionary and adaptive capacity of poxviruses, one of the potential triggers for the emergence of this epidemic was the change in the status of orthopoxvirus vaccination and eradication programs. This epidemic outbreak of HMPX spread worldwide, with a notable frequency in Europe, North America, and South America. Due to these particularities, the objective of the present study was to assess and compare cases of HMPX in these geographical regions through logistic and Gompertz mathematical modeling over one year since its inception. We estimated the highest contagion rates (people per day) of 690, 230, 278, and 206 for the world, Europe, North America, and South America, respectively, in the logistic model. The equivalent values for the Gompertz model were 696, 268, 308, and 202 for the highest contagion rates. The Kruskal–Wallis Test indicated different means among the geographical regions affected by HMPX regarding case velocity, and the Wilcoxon pairwise test indicated the absence of significant differences between the case velocity means between Europe and South America. The coefficient of determination (*R*^2^) values in the logistic model varied from 0.8720 to 0.9023, and in the Gompertz model, they ranged from 0.9881 to 0.9988, indicating a better fit to the actual data when using the Gompertz model. The estimated basic reproduction numbers ($R_{0}$) were more consistent in the logistic model, varying from 1.71 to 1.94 in the graphical method and from 1.75 to 1.95 in the analytical method. The comparative assessment of these mathematical modeling approaches permitted the establishment of the Gompertz model as the better-fitting model for the data and the logistic model for the $R_{0}$. However, both models successfully represented the actual HMPX case data. The present study estimated relevant epidemiological data to understand better the geographic similarities and differences in the dynamics of HMPX.

## 1. Introduction

The Monkeypox (also known as Mpox) virus (MPXV) is a zoonotic virus with similar clinical features to smallpox. MPXV belongs to the family *Poxviridae*, and the viral particle is a brick-shaped enveloped virion, 150–300 nm in size, that contains a double-stranded D.N.A. genome of 200 kbp on average [1]. Although the source of MPXV is among animals, it is still undetermined whether the viral host reservoirs and infection occur in species such as mice, rats, rabbits, hamsters, monkeys, humans, prairie dogs, woodchucks, jerboas, and porcupines [2].

The virus was initially isolated in 1958 from vesiculopustular lesions found in fever monkeys (Java macaques) in Denmark. However, the first recorded human Monkeypox (HMPX) infection occurred in 1970 in the Democratic Republic of the Congo, followed by sporadic outbreaks in eight African countries between 1970 and 1999, resulting in approximately 923 HMPX cases [3]. The first instance of HMPX outside Africa was reported in 2003 in the United States, with 47 cases. Between 2000 and 2020, a total of 20,237 cases of HMPX were reported across 16 countries [4]. Although MPXV had been primarily confined to African countries with occasional outbreaks elsewhere, in May 2022, a case of HMPX was documented in the United Kingdom. From that point until February 2023, the virus has rapidly spread to over 100 countries, leading to a total of 85,536 confirmed cases, with most cases concentrated in Europe, the U.S.A., and South America [5].

MPXV zoonotic transmission occurs through direct contact or consumption of infected animals. Human-to-human transmission usually takes place through indirect contact with respiratory secretions, skin lesions, or contaminated objects. However, direct contact remains a well-known risk factor for transmission [6]. MPXV infection causes a self-limiting disease with an incubation period of 4–14 days, and it is characterized by headaches, malaise, backache, fatigue, lethargy, and a low-grade fever. The vesiculopustular rash on the face and trunk appears 12–26 days after exposure, and the worst clinical outcomes, such as bronchopneumonia, encephalitis, and visual loss, are expressed in immunocompromised patients [6].

Since eradicating smallpox using the vaccinia virus in 1980, nearly four decades have passed without any orthopoxvirus vaccination programs. Consequently, discontinuing smallpox vaccination may have contributed to a reduction in, or even loss of, herd immunity against HMPX, potentially leading to an increase in the spread of the virus [7]. At present, the treatment for HMPX is primarily supportive, and antiviral medications like tecovirimat, cidofovir, and brincidofovir come with serious adverse effects. Additionally, only three FDA-approved vaccines have shown efficacy in clinical trials; however, there are currently no available data on their real-world effectiveness [8]. The long-term and rapid transmission in non-endemic regions worldwide has raised concerns about the potential evolution of MPXV into a more lethal pathogen. Moreover, the lack of treatment emphasizes the need for strategies to enhance epidemiological tracking and reckoning [9].

Since the 1920s, mathematical modeling approaches have been developed to understand dynamic growth and viral transmission patterns [10]. Logistic-regression-based models have been proposed for detecting and predicting epidemiology patterns in COVID-19, showing similar results between the dynamics of the virus in a real scenario and those calculated by the model [11,12,13]. In mathematical modeling based on a differential equation, along with the logistic model, there is another widely used model in population growth dynamics: the so-called Gompertz model, which has been widely used in tumor and epidemiological growth. Gompertz initially proposed this model in 1825 to study mortality in human populations. Since it was used by Casey in 1934 for the adjustment of tumor growth curves [14,15], its use in mathematical oncology has been ubiquitous because the solution curve of the differential equation used in the Gompertz model gives us the ability to model a saturated growth with a nonsymmetric inflection point compared to the logistic model whose sigmoid curve is symmetrical [16,17]. In epidemiology, it has been used along with other models such as the generalized logistic, Von Bertalanffy, and Richards, among others, for the adjustment of curves showing the population of people affected by COVID-19, for example [18,19].

Infection with the MPXV has previously been modeled using systems of ordinary first-order differential equations [20,21] and fractional order [22,23], which have considered both interaction with a sink for zoonotic transmission (rodents) as well as dissemination among the human population, and in some cases isolation of the sick and vaccination which provides permanent immunity have been also considered. Although models based on systems of differential equations provide a much more detailed explanation of the mechanisms of population propagation and allow us to simultaneously evaluate several epidemiological populations in addition to those infected as susceptible, latent, or recovered, models that characterize a single population, have been shown to fit well with the data in some studies [18,24,25].

This study aimed to assess and compare cases of HMPX in distinct continental regions through logistic and Gompertz differential equations over 12 months of the epidemic. In addition, we estimated the primary reproduction number for each model.

## 2. Materials and Methods

### 2.1. Data Collection

The primary dataset used to analyze Monkeypox infections in the present study was obtained from the World Health Organization’s (WHO) comprehensive report on global trends in Monkeypox for 2022–2023 [26]. The data variable under investigation pertained to the aggregate number of cases or infections, wherein “total cases” was defined as the sum of confirmed Monkeypox cases within the specified time frame, from 1 May 2022 to 30 April 2023 (Table S1).

To elucidate the epidemiological landscape of Monkeypox, a graphical representation was employed to illustrate the temporal evolution of the disease across the world and the continents with the highest incidence rates, namely Europe, North America, and South America. A comprehensive global overview was presented, treating it as a single entity for the specified time interval.

### 2.2. Mathematical Modeling

The mathematical modeling of the Monkeypox time series considering the variable of cases (or diagnosed infected people) was carried out using the logistic regression and the Gompertz function. These models use the sigmoid function to describe the growth of a variable with slower speeds at the beginning and end of a period.

The R programming language within the R Studio integrated development environment (IDE) incorporates various packages, including, but not limited to, tidyverse and ggplot2, as outlined in subsequent sections. These packages were used to visualize and model the results obtained [27].

#### 2.2.1. Logistic Model

The foundation of this model was rooted in the empirical modeling framework posited by Bronshtein and Semendiaev, and it was derived as an extension of the Verhulst–Pearl logistic model [28,29]. In the context of this research, this model was employed to assess and project the temporal patterns of Monkeypox cases within specific geographical regions, namely the world, Europe, North America, and South America.

The mathematical expression used to quantify the temporal dynamics of Monkeypox within these defined populations can be characterized as a logistic dispersion, and it is formulated as follows:

(1) $$N=\frac{M}{\left(1+Q\times e^{-k\times t}\right)}$$

In this mathematical representation, the symbol “*M*” signifies the maximum capacity for the occurrence of cases, “*Q*” denotes a pre-established constant, “*k*” represents a factor of proportionality, “*t*” signifies the elapsed time measured in days, and “*N*” represents the count of observed cases.

The formula utilized to calculate the maximum capacity “*M*” for the three distinct events necessitates the consideration of three independent stochastic variables, along with their associated dependent values retrieved from the dataset. This computation is performed according to the following mathematical expression [13]:

(2) $$M=\frac{A\times B-I^{2}}{A+B-2I}$$

The initial value, denoted as “*A*”, corresponds to the dependent variable at the inflection point of the independent variable “*t*_1_”. If the computed inflection point (mean value) is not a whole number, it is rounded to the next highest available integer value. This rounding rule will be used similarly for the following parameters and will include any linked value. The second value, designated as “*B*”, represents the dependent variable value corresponding to the final value of the independent variable “*t*_2_”. The third value, denoted as “*I*”, is associated with the dependent variable value related to the semi-sum of the independent variables “*t*_1_” and “*t*_2_,” expressed as “*t*_3_ = (*t*_1_ + *t*_2_)/2”. Subsequently, the ascertained value of “*M*” is inserted into the logistic model. The logistic model is then subjected to mathematical linearization, and the least squares method is employed to achieve the following form: $ln\left(\frac{M}{N}-1\right)=ln\;Q+k\times t$; a linear equation: y=A+Cx, where $y=ln\left(\frac{M}{N}-1\right)$, x=t, and A=ln Q.

Executing the statistical procedure of linear regression involves inputting paired data points (*x*, *y*) $\left[t,ln\left(\frac{M}{N}-1\right)\right]$, and upon entering all the data pairs to determine the values of *ln Q* and *k*, where *k* represents the slope of the linear equation (specifically, the ‘*C*’ coefficient in the equation: y=A+Cx, with A being lnB and thus, $Q=e^{A}$. This is achieved by deriving Equation (1), leading to the establishment of Equation (3). Equation (2) is then employed to ascertain the maximum possible number of infected individuals (*M*), a crucial value for subsequent calculations. To gauge the incidence rate of Monkeypox cases within the specified populations, we deduce the established logistic model, characterized by the following mathematical representation:

(3) $$\frac{dN}{dt}=\left[\frac{M\times Q\times k\times e^{-k\times t}}{{\left(1+Q\times e^{-k\times t}\right)}^{2}}\right]$$

To ascertain the critical time point, denoted as (*t*_c_), corresponding to the moment when the count of Monkeypox cases reaches its peak, we derive Equation (3), equal it to zero, and subsequently solve for (*t*_c_):

(4) $$t_{c}=-\frac{1}{k}\times ln\left(\frac{1}{Q}\right)$$

#### 2.2.2. Gompertz Model

The Gompertz model assumes that a population’s growth rate is density-dependent, that is, that the number of individuals in a later instant depends on the number of individuals previously, and the higher the initial number of individuals, the higher their growth rate will be. It is also part of the model’s formalized ordinary differential equations, whose solutions are sigmoid functions and, in the particular case that we present, depend on three parameters for further adjustment, and whose main characteristic is that the turning point of the curve is located before the midpoint of the curve, which gives it an asymmetrical aspect, meaning it can reflect processes where exponential growth occurs in early stages of the epidemic and then slows down [14,24].

The Gompertz differential equation can be posed as a modification of the logistic equation, given as $\frac{dN}{dt}=rN\left(1-\frac{\ln\left(N\right)}{\ln\left(\alpha \right)}\right),$ where is the infection rate (day^−1^), *α*, is the maximum cumulative number of infected people in each region, and *N* = *N*(*t*) is the cumulative number of infected from the onset of the epidemic versus time *t* (in days). Rewriting the equation, we produce: $\frac{dN}{dt}=rN\left(1-\frac{\ln\left(N\right)}{\ln\left(\alpha \right)}\right)=rN\left(\frac{\ln\left(\alpha \right)-\ln\left(N\right)}{\ln\left(\alpha \right)}\right)=\frac{r}{\ln\left(\alpha \right)}N\cdot ln\left(\frac{\alpha }{N}\right);$ by making a change in variable $\gamma =\frac{r}{\ln\left(\alpha \right)}$, we can express: $\frac{dN}{dt}=\gamma \cdot N\cdot ln\left(\frac{\alpha }{N}\right),$ where *γ* is the constant of proportionality related to the growth rate of the epidemic. Therefore, we can present the initial value problem as:

(5) $$\frac{dN}{dt}=\gamma \cdot N\cdot ln\left(\frac{\alpha }{N}\right),\;\;t\left(0\right)=t_{0},\;N\left(t_{0}\right){=N}_{0},$$

where *t*_0_ is a point of reference from the beginning of the epidemic and *N*_0_ > 0 is the number of infections accumulated at the beginning of the infection over time *t*_0_. The analytical solution of the differential Equation (5) is as follows:

(6) $$N\left(t\right)=N=\alpha \cdot e^{-\ln\left(\frac{\alpha }{N_{0}}\right)\cdot e^{-\gamma t}}$$

Making an additional variable change we produce: $\beta =\ln\left(\frac{\alpha }{N_{0}}\right),$ where *β* is a parameter that controls how quickly the population approaches *α*. The higher the *β*, the faster the population will approach the maximum asymptotic value of *α*. From which we can express the Gompertz function as:

(7) $$N\left(t\right)=N=\alpha e^{-\beta e^{-\gamma t}}\;$$

The function found in (7) will be our curve, to which we will adjust the selected data to apply the same methodology to determine parameters as in the logistic model. The value of *α* was calculated using the mean values described by Bronshtein and Semendiaev, while the parameters *β* and *γ* were obtained with linear regression [30].

Taking natural logarithm to (7), we obtained,

(8) $$N\ln\left(N\right)=ln\alpha -\beta e^{-\gamma t}\;\ln\left(N\right)-ln\alpha =-\beta e^{-\gamma t}$$

Making the following variable change, it becomes,

(9) $$y=\ln\left(N\right),\;c=ln\alpha \;y-c=-\beta e^{-\gamma t}$$

And linearizing produces,

$$\ln\left(y-c\right)=\ln\left(-\beta \right)-\gamma t$$

If we have three points representing the epidemiological data of accumulated infected: (*t*_1_, *y*_1_), (*t*_2_, *y*_2_), (*t*_3_, *y*_3_), we can estimate the parameter *c* [30].

(10) $$c=\frac{y_{1}y_{2}-y_{3}^{2}}{y_{1}+y_{2}-2y_{3}}$$

By reversing the variable changes in (8) and taking the same three points as those considered for the logistics model (*t*_1_, *A*), (*t*_2_, *B*), (*t*_3_, *I*), where *t*_3_ = (*t*_1_ + *t*_2_)/2, which we must replace in (10), we can calculate the value of *α*:

(11) $$ln\alpha =\frac{\ln\left(A\right)\ln\left(B\right)-\ln^{2}\left(I\right)}{\ln\left(A\right)+\ln\left(B\right)-2\ln\left(I\right)}\;\alpha =e^{\left(\frac{\ln\left(A\right)\ln\left(B\right)-\ln^{2}\left(I\right)}{\ln\left(A\right)+\ln\left(B\right)-2\ln\left(I\right)}\right)}$$

On the other hand, from expression (8) we must:

$$ln\alpha -\ln\left(N\right)=\beta e^{-\gamma t}$$

$$\ln\left(\frac{\alpha }{N}\right)=\beta e^{-\gamma t}$$

And by linearizing, we produce:

(12) $$\ln\left(\ln\left(\frac{\alpha }{N}\right)\right)=ln\beta -\gamma t$$

From the second linearization Equation (12), and by applying the least squares method approach with the line Y=a+bX, where $Y=\ln\left(\ln\left(\frac{\alpha }{N}\right)\right),\;X=t,\;b=-\gamma$, and a=ln(β), it is possible to find the values of the parameters *α* and *γ* of the Gompertz equation.

To estimate the parameters through linear regression, the data were tabulated as ordered pairs (*t*, *N*), and the values of $Y=ln\left(\ln\left(\frac{\alpha }{N}\right)\right)$ and *X* = *t* were calculated directly from the estimated line, the values of γ=−b, and a=ln(β), then $\beta =e^{a}$.

To estimate the rate of cases due to Monkeypox in all study populations, the Gompertz function was derived, and its differential equation was found:

$$\frac{dN}{dt}=\alpha \beta \gamma e^{-\beta e^{-\gamma t}-\gamma t}$$

In order to determine the critical value (*t_c_*), which represents the maximum value of the daily cases observed in the data, the second derivative was calculated, which has the form:

(13) $$\frac{d^{2}N}{dt^{2}}=\alpha \beta \gamma e^{-\beta e^{-\gamma t}-\gamma t}\left(\beta \gamma e^{-\gamma t}-\gamma \right)$$

Moreover, expression (13) was equal to zero, which geometrically represents the time coordinate of the inflection point of the Gompertz curve of the accumulated cases (data), obtaining:

$$t_{c}=\frac{\ln\left(\beta \right)}{\gamma }$$

If the value obtained is not an integer, the following integer value is selected (through rounding), and then this value serves as a reference to find the date and maximum daily infection rate value in the data.

### 2.3. Statistical Analysis

In the R programming language (version 4.2.3), we utilized additional packages within the R Studio integrated development (IDE) environment, namely Nortest and Stats. The Nortest package comprises a set of R functions tailored for executing normality tests, while the Stats package encompasses a range of R functions dedicated to statistical tests and comprehensive data analysis. Additionally, for model validation, we employed the lmtest package to conduct the Breusch–Pagan test.

#### 2.3.1. Normality Tests for the Variable Cases

The Monkeypox Total Cases variable underwent a segmentation process for statistical analysis, was stratified according to the respective population groups under investigation, and categorized by geographical region (i.e., world, Europe, North America, South America). This segmentation was performed to determine the most appropriate statistical tests to be subsequently applied to the dataset for both the logistic model and the Gompertz model.

Hypothesis tests were conducted to assess the normality of the data within each group. These tests were designed to ascertain whether the data distribution in each group adheres to a normal distribution. The outcome of these tests is represented by a *p*-value, which quantifies the probability of observing a data distribution similar to or deviating further from normality. This is carried out assuming the null hypothesis posits that the variable conforms to a perfectly normal distribution within the population [31]. In cases where the p-value exceeds the predetermined significance level, inadequate evidence exists to reject the null hypothesis. This suggests that the variable follows a normal distribution [31].

The Kolmogorov–Smirnov test was employed because the dataset within each population group exceeded a sample size of *N* > 50. This test used the lillie—test function from the Nortest package for each population.

Hypothesis Test:

**H0:**  *The data follows a normal distribution.*

**H1:**  *The data does not follow a normal distribution.*

A significance level (α) of 0.05 was established.

#### 2.3.2. Kruskal–Wallis Test for Monkeypox Cases Velocity

Based on the normality test results, the Kruskal–Wallis test was conducted for both the logistic and Gompertz models. This non-parametric test assesses differences among three or more independent groups sampled from a single non-normally distributed continuous variable [32]. To perform the test, the Kruskal.test (case velocity~geographic region, data = monkeypox) function was used.

Hypothesis Test:

**H0:**  *No significant differences among the means of the populations under study exist.*

**H1:**  *At least one mean significantly differs from the other populations.*

A significance level (α) of 0.05 was established.

#### 2.3.3. Post-Hoc Test: Pairwise Wilcoxon Test for Monkeypox Cases Velocity

Based on the results of the Kruskal–Wallis test, a post-hoc test was conducted to determine which means exhibited significant differences for both the logistic model and the Gompertz model. As a post-hoc test for the Kruskal–Wallis test, multiple non-parametric pairwise comparisons are typically performed, often using the pairwise Wilcoxon test [33]. To conduct the test, the pairwise. wilcox.test (case velocity~geographic region, data = monkeypox) function was employed.

Hypothesis Test for each combination:

**H0:**  *No significant differences exist between the means of populations X and Y.*

**H1:**  *There are significant differences between populations X and Y.*

X and Y represent any pair of populations analyzed by the Wilcoxon test.

A significance level (α) of 0.05 was established.

#### 2.3.4. Multiple Linear Regression Analysis for Monkeypox Cases Velocity

Additionally, a multiple linear regression test was conducted to assess the effect of each geographic region on the velocity of Monkeypox cases and to validate the conclusions drawn from the Kruskal–Wallis and Wilcoxon tests for the logistic model and the Gompertz model. Time and geographic region were used as independent variables to determine if they are explanatory in the multiple linear regression model [34]; in other words, if they affect the velocity of Monkeypox cases. The lm(case velocity~time + geographic region, data = monkeypox) function was utilized to perform the test.

Hypothesis Test for each geographic region:

**H0:**  *Geographic region does not affect the velocity of Monkeypox cases.*

**H1:**  *Geographic region affects the velocity of Monkeypox cases.*

The geographic regions are Europe, North America, and South America.

A significance level (α) of 0.05 was established.

#### 2.3.5. Comparison of Modeled Variable against Real Data

To assess the degree to which the mathematical models align with the empirical data, we computed the coefficient of determination denoted as *R*^2^, as elaborated by Schober, et al. [35], for both the logistic model and the Gompertz model. *R*^2^ indicates the proportion of the total variability observed in the response variable that the model can elucidate. A higher *R*^2^ value, approaching 1, signifies a robust model fit, implying that the model can account for a substantial portion of the response variable’s variability. Conversely, a lower *R*^2^ value, approaching 0, implies that the model inadequately explains the variability inherent in the response variable.

To calculate *R*^2^, we employed the subsequent mathematical formula: *R*^2^ = 1 − (SSR/SST), where S.S.R. (Sum of Squares Residual) represents the summation of squared discrepancies between the predicted values derived from the model and the actual values of the response variable. At the same time, S.S.T. (Sum of Squares Total) signifies the summation of squared discrepancies between the actual values of the response variable and its mean.

Additionally, the Akaike information criterion (A.I.C.) was used as a second metric to analyze the goodness of fit of our models to the actual data. This criterion evaluates the quality of the adjustment of the models considering the number of parameters used in each model and the number of observations made, choosing the best model that minimizes the A.I.C. index. This index is calculated as [18]:

$$AIC=n\cdot \ln\left(\frac{\sum_{i=1}^{n}{\left(y_{i}-{\hat{y}}_{i}\right)}^{2}}{n}\;\right)+2\cdot p$$

where *n* is the amount of data analyzed (in our case, 365 days), *p* is the number of parameters of our model (*p* = 3, for both models), and the difference in residuals squared is given between the observed values (actual data) *y*_i_ and the predicted values for each of the models *ŷ*_i_. This index was estimated after tabulating data accumulated for the infected population (actual data, estimated data from the logistic and Gompertz models). Three parameters were estimated for the logistics model (*M*, *A*, *k*) and the Gompertz model (*α*, *β*, *γ*).

### 2.4. Estimation of the Basic Reproduction Number

The primary reproduction number is a relevant epidemiological parameter at the beginning of an epidemic outbreak; it indicates the number of secondary infections that can occur when an infected individual is in contact with a population susceptible to infection. Its epidemiological interpretation means that, for $R_{0}$ > 1, the disease will spread, while for $R_{0}$ < 1, the outbreak will tend to limit itself. Aware of the importance of this epidemiological parameter, we employed two different approaches to estimate it using the data obtained from the two mathematical models in the present study. This was carried out to evaluate which of these approaches could better reflect a real scenario. In our case, we estimate $R_{0}$ values for both models using two empirical methods. To implement them, we need to know the infectious time and incubation period (pre-infectious period) of the disease. Then, we estimate the value of $R_{0}$ using the following formula [36]:

(14) $$R_{0}=(1+r\cdot D)(1+r\cdot D^{\prime })$$

where *r* is the growth rate of the epidemic, D is the average time of infection, and $D^{\prime }$ is the average incubation time. This approach is valid when we assume that D and $D^{\prime }$ follow an exponential distribution, and when D and $D^{\prime }$ are relatively short compared to each other.

The first methodology presented in [36] is a graphical method by which we tabulate the first 26 days of the epidemic (considering the quasi-exponential behavior in the early stages of the epidemic outbreak), concerning the natural logarithm of the accumulated data, and using linear regression, we estimate the growth rate of the epidemic “*r*” (intrinsic growth of the infected population per each model) as the slope of the straight. The obtained values for *r* are then utilized in Equation (14).

The second methodology used is an analytical approach, since at the beginning of an epidemic, the number of infected persons accumulated is much lower than their maximum capacity, *N* ≪ *N_max_*; therefore, it is possible to approximate a logistic or Gompertz function for exponential growth.

In the case of the differential logistic equation $\frac{dQ}{dt}=kQ\left(1-\frac{Q}{M}\right)$, when Q<M, Q/M tends to zero, and therefore, dQ/dt=rQ, whose solution is $Q(t)=Q\left(0\right)e^{kt}$, where $r_{logistic}=k$ is the intrinsic growth rate of the epidemic “*r*”.

For the Gompertz model $\frac{dN}{dt}=\gamma Nln\left(\frac{\alpha }{N}\right)$, we can approximate ln(α/N) by using a Taylor series: ln(α/N)=ln(α)−N/α. So, dN/dt=γN(ln(α)−N/α), and when N<α, then dN/dt=γln(α)N. The solution is $N(t)=N\left(0\right)e^{\gamma ln\left(\alpha \right)t}$, where $r_{gompertz}=\gamma ln(\alpha )=r$. The obtained values for *r* are then utilized in Equation (14).

## 3. Results

### 3.1. Epidemiological Panorama of Monkeypox

**Figure 1:**
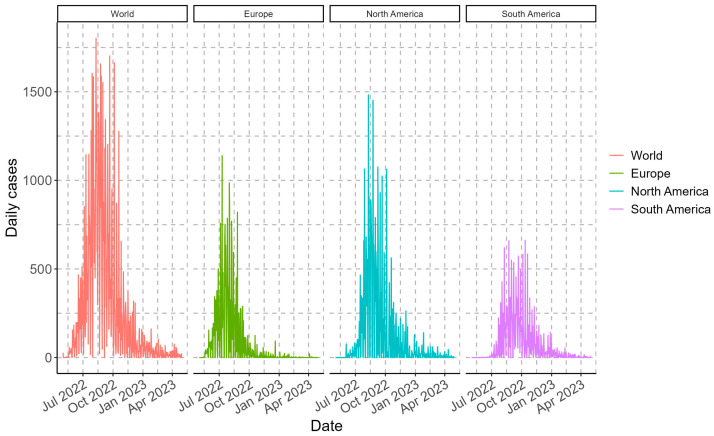
Epidemiological overview of daily Monkeypox daily cases for the different geographic regions analyzed in one year.

The Monkeypox outbreak that began in May 2022 accumulated more than 87 thousand cases worldwide until this study’s analysis date (30 April 2023). Worldwide, the maximum values of daily cases were recorded between 24 August and 5 October 2022, exceeding 1650 cases per day. In the case of the continents, the maximum case values were recorded in 2022, between 10 July to 2 August (for Europe), 10 to 24 August (for North America), and between 9 August to 12 October (for South America), with maximum values exceeding 860, 1450, and 620 cases per day, respectively (Figure 1). Consequently, the accumulated case values as of the cut-off date were more than 87.2 thousand (world), 25.6 thousand (Europe), 36.9 thousand (North America), and 22.3 thousand (South America).

### 3.2. Mathematical Modeling

**Figure 2:**
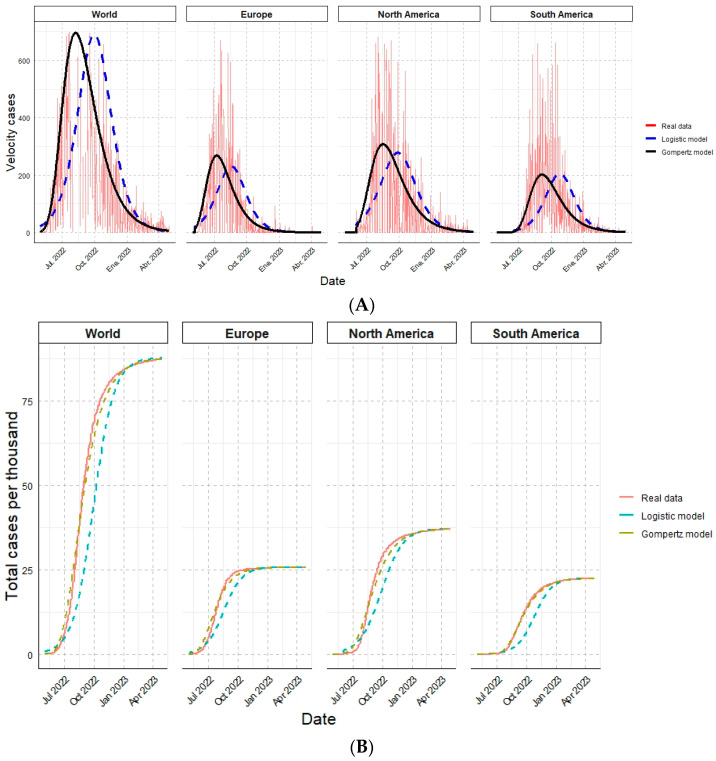
Epidemiological overview of Monkeypox cases for the different geographic regions analyzed in one year, comparing the actual data against the mathematical models. (**A**) Velocity of cases. (**B**) Total cases per thousand.

The mathematical models (logistic and Gompertz) were applied to each region (world, Europe, North America, and South America) independently. The results obtained are shown below (Table 1):

Finally, the results estimated by the logistic and the Gompertz models were compared with the actual data and observed simultaneously. The contagion rate of Monkeypox cases by each analyzed geographic region (Figure 2A) and the cumulative cases (Figure 2B) are shown:

### 3.3. Statistical Analysis

#### 3.3.1. Normality Tests for the Variable Cases

The data from the populations (world, Europe, North America, and South America) for the modeled data did not exhibit a normal distribution. The *p*-value for each population (Table 2) was less than 0.05, indicating the rejection of the null hypothesis and establishing that the data does not follow a normal distribution.

#### 3.3.2. Kruskal–Wallis Test for Monkeypox Cases Velocity

The Kruskal–Wallis mean comparison test was conducted to assess whether there are significant differences in the Monkeypox case velocities among each geographic region for the logistic and Gompertz models. The obtained *p*-values were 1.55 × 10^−41^ and 2.71 × 10^−42^, respectively, rejecting the null hypothesis and indicating that at least one population whose mean significantly differs from the means of the other populations under study for the logistic model and the Gompertz model.

#### 3.3.3. Post-Hoc Test: Pairwise Wilcoxon Test for Monkeypox Cases Velocity

The post hoc test, the Wilcoxon pairwise test, was performed to evaluate which mean or means present significant differences compared to the other populations for both the logistic and Gompertz models. The obtained *p*-values (Table 3) were mostly less than 0.05, rejecting the null hypothesis and indicating significant differences among the following populations: world and Europe, World and North America, world and South America, Europe and North America, North America, and South America for both the logistic model and the Gompertz model. However, the populations of Europe and South America have a *p*-value of 0.894 for the logistic model and 0.546 for the Gompertz model, which is greater than 0.05, hence accepting the null hypothesis and indicating that there are no significant differences between the means of both populations for both models.

#### 3.3.4. Multiple Linear Regression Analysis for Monkeypox Cases Velocity

The multiple linear regression test was conducted to determine and validate whether geographic region affects the Monkeypox case velocity for both the logistic and Gompertz models. The obtained p-values for each geographic region (Table 4) were less than 0.05, rejecting the null hypothesis and indicating that there is an effect of geographic region on the Monkeypox case velocity, supporting the results obtained in the Kruskal–Wallis and Wilcoxon tests.

#### 3.3.5. Comparison of Modeled Variable against Real Data

The comparison of actual data with the data obtained from the mathematical model for the Monkeypox case velocity by geographic region resulted in determination coefficients (*R*^2^) for both the logistic model and the Gompertz model (Table 5):

These values indicate that both mathematical models firmly represent the actual data for all variables. However, it is essential to emphasize that the Gompertz model would be better because the coefficients obtained are more significant than the logistic model and close to one. In particular, the Gompertz model applied to South America showed the highest value among all (0.9988).

The adjustment analyses’ results by calculating the coefficient of determination and the Akaike information criterion (A.I.C.) are also shown in Table 5:

A higher coefficient of determination and a lower value of the A.I.C. index for the Gompertz model indicate a better fit to our data than the logistic model.

### 3.4. Estimation of the Basic Reproduction Number

**Figure 3:**
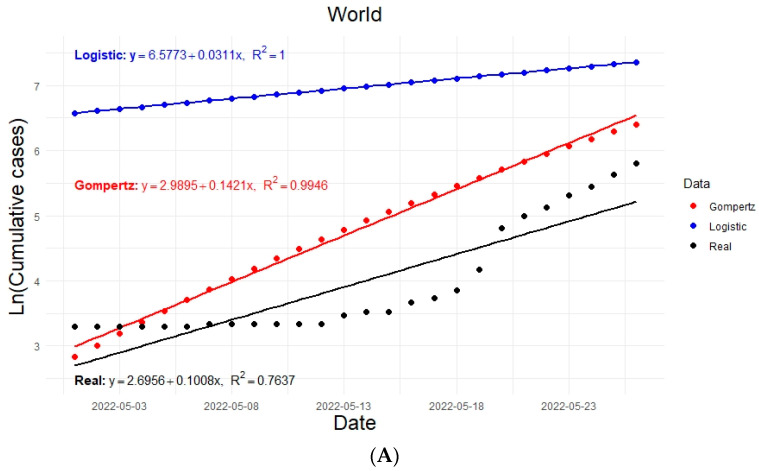

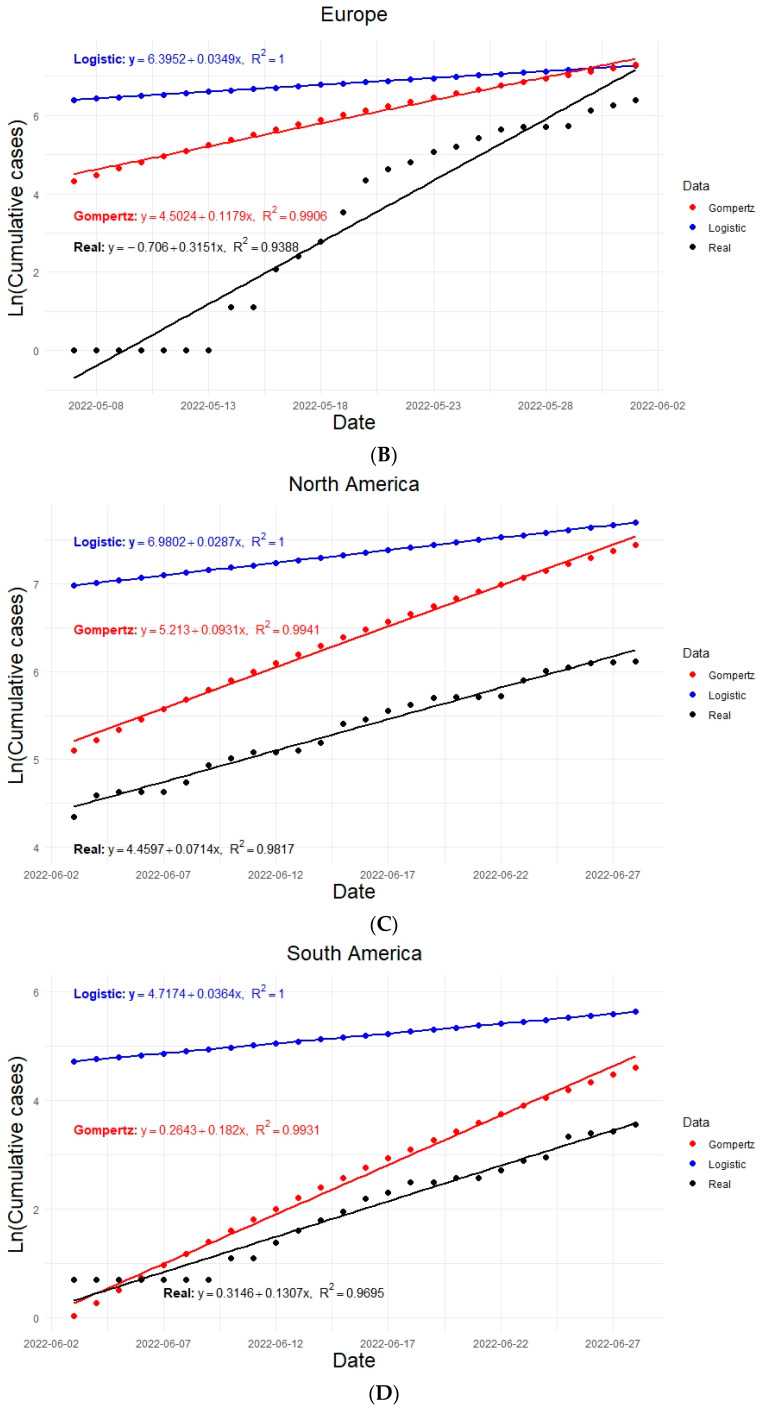
Linear regression curves of the actual and modeled data considering 26 days of exponential growth and taking the natural logarithm of the accumulated data for the infected population shown from top to bottom for (**A**) world, (**B**) Europe, (**C**) North America, (**D**) South America.

Graphically, it was possible to estimate the growth rates of the epidemic for each model in each region ($r_{logistic}$, $r_{gompertz}$). The estimated regression lines are shown in Figure 3. The growth values of the epidemic are considered as the slopes of the regression lines in each case; for example, for the world: $r_{logistic}=0.0311$ and $r_{gompertz}=0.1421$.

While the analytical approach also found values of ($r_{logistic}=k$, $r_{gompertz}=\gamma \cdot ln(\alpha )$), these are shown in Table 6:

The calculations for $R_{0}$ assumed that the pre-infectious period is the average incubation period, $D^{\prime }=9.1$ days, while the infectious period is the mean generation period, D=12.5 days [37]. For example, to calculate $R_{0}$ for the world with the r_logistic obtained using the graphical method: $R_{0}=\left(1+0.0311\times 9.1\right)\times \left(1+0.0311\times 12.5\right)=1.78178$. The estimated $R_{0}$ values using both methodologies are shown in Table 7.

## 4. Discussion

This study was conducted one year after the HMPX epidemic outbreak in different geographic regions, and through comparative mathematical modeling, the epidemiological dynamics of cases were assessed.

Poxviruses are pathogens closely linked to the history of humanity [38,39]. Despite evolving slowly due to their sizeable double-stranded D.N.A. genome, poxviruses are highly adaptable and can undergo genotypic and phenotypic alterations to adapt and thrive in new hosts [40,41,42]. Furthermore, their genomic architecture can shape their evolution and sometimes interact with other viruses [41,43].

The HMPX outbreak presents some peculiar and intriguing features in the field of virus epidemiology. First and foremost, it is an epidemic occurring within an ongoing pandemic (COVID-19) caused by the SARS-CoV-2 virus [44,45]. Secondly, despite its primary association with primates, it can infect various animal and human species [2]. Unlike the outbreaks in humans in past decades [4], the 2022 epidemic outbreak constitutes the first significant case of global dispersion, affecting several countries and continents [5]. Third, the global discontinuation of vaccination programs against orthopoxviruses (i.e., vaccinia virus vaccine) after the eradication of smallpox potentially correlates with the re-emergence of this virus [7], implying the need to review global vaccination strategies to prevent or control new outbreaks of HMPX.

Mathematical models play a pivotal role in the anticipatory analysis of the propagation of infectious diseases, providing a crucial foundation of information for decision-makers in public health and government policy. In the present study, in addition to addressing different data, scales, and geographical spaces, we aimed to include comparative approaches and novel methodologies. While many epidemiological studies have developed compartmental mathematical models to understand the infectious dynamics of HMPX [20,21,22,23,46], there are not many studies that have attempted to explain the dynamics using a single differential equation for the infected population in HMPX. In this sense, our study provides a primary approximation model for HMPX with two large and well-studied mathematical models, namely the logistic and Gompertz models. These models have proven to be useful in describing infectious dynamics in many cases [18,19]. Furthermore, we implemented the methodology presented by Bronshtein and Semendiaev [30] in the case of fitting the data using three points for the Gompertz model. Additionally, we calculated $R_{0}$ using an empirical and analytical approach with real data in models based on a single ordinary differential equation.

Performing mathematical modeling of HMPX cases using two models simultaneously allows us to establish comparisons to determine the one with the best fit. Thus, based on the coefficient of determination values, the Gompertz model fits our data better than the logistic model. This is because, in the daily data (velocity of cases), a slightly asymmetrical distribution is observed, with the highest rates of contagion occurring at the beginning of the epidemic. Geometrically, this represents that the tipping point (maximum speed, corresponding to the maximum number of daily recorded infections in the data) occurs before the midpoint of the accumulated infection curve.

This characteristic and a better adjustment to data with these features have been determined in several epidemiological studies, in which models based on differential equations are also compared [18,19]. Although there are equivalent ways of writing the Gompertz function with three parameters, it is recommended to use the form in which the time coordinate is explicitly presented at the inflection point $t_{c}$ [14,24]. The methodology used in this study enables us to estimate the critical time $t_{c}$ based on the presented function. It also allows us to highlight the relationship between the rates of the epidemic spread r (infection rate, epidemic growth rate, or population growth rate of infected in each region), the maximum cumulative amount α, and the initial number of infected $N_{0}$ concerning the parameters of the Gompertz curve. We determined that $\beta =\ln\left(\frac{\alpha }{N_{0}}\right)$ and $\gamma =\frac{r}{\ln\left(\alpha \right)}$. In the case of β, this parameter controls the speed with which, given an initial amount of accumulated infections, these approach their maximum accumulated amount. The more significant the difference between α and $N_{0}$, the faster the infected population will grow. This is deduced by taking the limit in Equation (7) to: $\underset{\beta \to \infty }{\lim}N\left(t\right)=\underset{\beta \to \infty }{\lim}\alpha e^{-\beta e^{-\gamma t}}=0$. Therefore, in Equation (5): $\gamma \cdot N\cdot ln\left(\frac{\alpha }{N}\right)\to 0$, when N→α. On the other hand, for γ, we can calculate the intrinsic growth rate for the infected population as $r=\gamma ln\left(\alpha \right).$ Finally, we can calculate $R_{0}$, one of the most relevant parameters in viral epidemiology.

In the Gompertz model, taking the logarithm of the equation results in a nonlinear equation on the logarithmic scale, given the presence of the natural logarithm function ln(α/N). Consequently, the relationship between the slope of the linear regression and $r_{gompertz}$ is not direct. It cannot be immediately obtained through the slope of the regression line on the logarithmic scale. In contrast, when we take the logarithm of the logistic model equation, a linear relationship emerges on the logarithmic scale. This allows the slope of the linear regression to directly provide an estimate of the intrinsic growth rate ($r_{logistic}$). We also observe discrepancies when determining $r_{gompertz}$ through the two methodologies. These discrepancies arise from the Gompertz function’s mathematical structure, which does not allow the determination of the growth rate through a single logarithm. Additionally, this value (the slope of the regression lines) is sensitive to the assumed amount of data as exponential growth. As a result of the above, significant discrepancies in the $R_{0}$ values estimated by the Gompertz model are noted in each method used to find $r_{gompertz}$. In any case, it is essential to mention that, the $R_{0}$ calculated from the Gompertz model is greater than that calculated by the logistic model. At the beginning of an outbreak, there is always a lack of data because the disease is unknown. The Gompertz model, being asymmetric, rises more quickly, leading to an $R_{0}$ that is always higher than the logistic model. However, these $R_{0}$ estimates should be viewed as initial and imprecise empirical references. They depend on factors such as periods of pre-infection and infection, and the method of calculating the rate of outbreak growth, which may vary significantly in each region and study population. Additionally, the values of the periods of infection and pre-infection (incubation) used for our calculations represent only average values within a wide range. Different ranges of values can be found in the literature [37,46,47].

For the logistic model, we note that when estimating $r_{logistic}$ with a small population Q compared to its carrying capacity *M*, its growth resembles exponential growth, and the growth rate is approximately constant. However, as the population approaches carrying capacity (Q approaches M), growth slows and approaches zero, otherwise known as the self-regulation effect of carrying capacity. Therefore, the logistic equation captures both the initial exponential growth and the eventual stabilization of the population as it approaches M. However, it is imperative to recognize the inherent limitations of logistic models, as they assume a uniform population and equivalent vulnerability to infection.

Both methods of estimating $r_{gompertz}$ are valid only for small values of N and for an initial growth phase. As the population grows and approaches carrying capacity (α), the behavior of the Gompertz equation differs significantly from exponential growth. Therefore, this approach adequately describes the initial behavior but does not represent the complete behavior of the Gompertz model.

Due to the methodologies used, the values of $R_{0}$ should be taken as illustrative and are presented to quantitatively show that $R_{0}>1$ in any case, and the epidemic outbreak, in general, occurs faster according to the Gompertz model, which fits better with the observed data in Figure 3. Here, we note that the slopes found are closer to those found in the actual data. However, the logistic model estimated consistent values of $R_{0}$ (through graphical and analytical approximations), and they were closer to those observed at the beginning of the epidemic [48].

Even though the differences mentioned above between the two models impacted the early (and better-adjusted) estimates of *t_c_* and the highest contagion rate for the Gompertz model, both models validly approximated the epidemiological behavior of HMPX, and how geographic region affects its contagion velocity. This allowed us to observe significant differences in Monkeypox case velocities among each geographic region in both models, except for the peer evaluation between Europe and South America, which exhibited similar behaviors (a flatter curve) compared to North America and the world, which presented more accentuated curves. All these mathematical estimates with an epidemiological relationship enable us to understand better the dynamics of HMPX and its potential preventive and clinical implications.

Historically, Monkeypox was a self-limiting disease. However, the recent outbreak has indicated a shift in its transmission patterns, especially among men who have sex with men (MSM) [45]. Clinical manifestations have evolved, with patients now exhibiting unusual symptoms such as proctitis, tonsillitis, and paraphimosis related to penile edema [49]. Amidst this health crisis, there is a silver lining in the antigenic similarity between the smallpox virus and the Monkeypox virus, allowing for the use of smallpox vaccines as a preventive measure against Monkeypox [50].

Strategies for managing and preventing Monkeypox are based on measures initially designed for smallpox protection. Smallpox is the only infectious disease that humanity has successfully eradicated, and the strategies employed could be applied to Monkeypox [51]. However, the challenge lies in understanding the Monkeypox virus and its transmission patterns and developing effective treatment and prevention strategies [52].

The recent Monkeypox epidemic has highlighted the virus’s adeptness at evading the host’s immune system. The virus has developed strategies to control the activation of antiviral T cells and the production of inflammatory cytokines. This immune evasion mechanism is crucial for the virus’s efficient spread within the infected host [53]. Like other poxviruses, the Monkeypox virus has evolved multiple mechanisms to evade the host’s immune system [54]. The discontinuation of smallpox vaccination, which provides cross-protection against Monkeypox, may have significantly contributed to the 2022 outbreak [7]. A study on the Jynneos vaccine and Monkeypox infection delves into the immune responses elicited, presenting fewer side effects than earlier smallpox vaccines [55]. It represents an example of potential improvements in vaccination strategies against HMPX and highlights the need to review current immunization programs.

With respect to the aforementioned information, the models used in the present study can consider the vaccination factor as a coefficient derived from the empirical efficacy values of a potential authorized vaccine against HMPX, or they can be used to simulate various vaccine scenarios. Alternatively, the vaccination factor could be simulated by implementing fractional order or compartmentalized models, as has been published for other viral diseases [56,57,58]. The logistic model is better suited to the initial and mid-stages of an epidemic, offering a symmetrical S-shaped curve that depicts consistent viral spread and carrying capacity. In contrast, the Gompertz model excels in later stages, capturing slowed disease spread with its asymmetrical S-shaped curve influenced by factors like immunity. Consequently, it can be deduced that the model choice depends on the epidemic phase, disease spread nature, and data fit. Both models share a sigmoid and bounded function, but the logistic model’s tipping point creates a symmetric curve, while the Gompertz model’s inflection point leads to asymmetry. Model selection hinges on observed data characteristics, considering the Gompertz model’s potential initial growth rate overestimation.

## 5. Conclusions

The present study demonstrates that the use of mathematical models based on single differential equations firmly represents the real data, as shown by the determination coefficients (*R*^2^) for both the logistic model and the Gompertz model. Consequently, this allows for the estimation of important epidemiological values such as *t*_c_, the contagion rate, and the basic reproduction number ($R_{0}$).

The case data in the selected geographic regions showed a non-normal distribution and the significant effect of the geographic region on Monkeypox case velocity. Furthermore, significant differences were observed between case rates in these regions, except for Europe and South America, which exhibited flatter curves.

Finally, due to the nature and symmetry (or asymmetry) of the compared models, it was observed that the Gompertz model better represented the real case data, while the logistic model allowed the estimation of consistent values of $R_{0}$ (through graphical and analytical approximations), which were closer to those observed at the beginning of the epidemic.

## Figures and Tables

**Table 1:** Estimated parameters for the logistic and the Gompertz models.

Model	Parameters	World	Europe	North America	South America
Logistic	Coefficient of Determination *R*^2^	0.8885	0.8720	0.9023	0.8774
Logistic	Critical time *t*_c_ (days)	152	108	117	144
Logistic	Date on *t*_c_	30 September 2022	23 August 2022	28 September 2022	25 October 2022
Logistic	*N_max_* on *t*_c_	43,648	13,926	18,515	11,160
Logistic	Highest contagion rate (people/day)	690	230	278	206
Gompertz	Coefficient of Determination *R*^2^	0.9952	0.9881	0.9900	0.9988
Gompertz	Critical time *t*_c_ (days)	100	62	75	94
Gompertz	Date on *t*_c_	9 August 2022	8 July 2022	17 August 2022	5 September 2022
Gompertz	*N_max_* on *t*_c_	32,656	9463	13,699	8264
Gompertz	Highest contagion rate (people/day)	696	268	308	202

**Table 2:** Results of the Normality Test.

Model	Geographic Region	*p*-Value	Hypothesis Testing	Interpretation
Logistic	World	6.39 × 10^−34^	Reject H0	Non-Normal Distribution
Logistic	Europe	4.11 × 10^−60^	Reject H0	Non-Normal Distribution
Logistic	North America	1.08 × 10^−32^	Reject H0	Non-Normal Distribution
Logistic	South America	1.68 × 10^−30^	Reject H0	Non-Normal Distribution
Gompertz	World	1.94 × 10^−39^	Reject H0	Non-Normal Distribution
Gompertz	Europe	3.77 × 10^−98^	Reject H0	Non-Normal Distribution
Gompertz	North America	5.37 × 10^−39^	Reject H0	Non-Normal Distribution
Gompertz	South America	2.87 × 10^−36^	Reject H0	Non-Normal Distribution

**Table 3:** Results of the post hoc test, Wilcoxon pairwise test.

Model	Geographic Region	World	Europe	North America
Logistic	Europe	3.71 × 10^−30^	-	-
Logistic	North America	3.92 × 10^−15^	8.65 × 10^−6^	-
Logistic	South America	6.23 × 10^−32^	0.894	1.73 × 10^−6^
Gompertz	Europe	3.48 × 10^−32^	-	-
Gompertz	North America	1.71 × 10^−15^	2.93 × 10^−6^	-
Gompertz	South America	5.17 × 10^−31^	0.546	5.15 × 10^−6^

**Table 4:** Results of the multiple linear regression analysis for Monkeypox case velocity.

Model	Geographic Region	*p*-Value	Hypothesis Testing	Interpretation
Logistic	World	<2.2 × 10^−16^	Reject H0	Significant effect
Logistic	Europe	0.001295	Reject H0	Significant effect
Logistic	North America	0.000108	Reject H0	Significant effect
Logistic	South America	9.74 × 10^−7^	Reject H0	Significant effect
Gompertz	World	<2.2 × 10^−16^	Reject H0	Significant effect
Gompertz	Europe	0.000314	Reject H0	Significant effect
Gompertz	North America	<2.2 × 10^−16^	Reject H0	Significant effect
Gompertz	South America	<2.2 × 10^−16^	Reject H0	Significant effect

**Table 5:** Adjustment values for models and actual data for each region studied.

Geographic Region	$R^{2}$–Logistic	$R^{2}$–Gompertz	A.I.C.–Logistic	A.I.C.–Gompertz
World	0.8885	0.9952	6836.8260	5669.1384
Europe	0.8720	0.9881	5783.6794	5066.7892
North America	0.9023	0.9900	6087.4334	5336.7998
South America	0.8774	0.9988	5869.6060	4229.3746

**Table 6:** Growth epidemic values are determined by each region by graphical and analytical approximation.

Geographic Region	Graphical $r_{logistic}$	Graphical $r_{gompertz}$	Analytical $r_{logistic}$	Analytical $r_{gompertz}$
World	0.0311	0.1421	0.0315	0.2458
Europe	0.0349	0.1179	0.0362	0.2893
North America	0.0287	0.0931	0.0300	0.2377
South America	0.0364	0.1820	0.0367	0.2454

**Table 7:** $R_{0}$ values are determined by each region by graphical and analytical approximation.

Geographic Region	Graphical Logistic $\;R_{0}$	Graphical Gompertz $\;R_{0}$	Analytical Logistic $\;R_{0}$	Analytical Gompertz $\;R_{0}$
World	1.78178	6.36625	1.79327	13.18179
Europe	1.89239	5.12781	1.93098	16.76913
North America	1.71361	3.99690	1.75038	12.56134
South America	1.93695	8.69906	1.94593	13.15080

## Data Availability

The primary dataset used to analyze Monkeypox infections in the present study was obtained from the World Health Organization’s (WHO) comprehensive report on global trends in Monkeypox for the year 2022–2023 [26] within the specified time frame, spanning from 1 May 2022, to 30 April 2023.

## Supplementary Materials

The following supporting information can be downloaded at https://www.mdpi.com/article/10.3390/vaccines11121765/s1, Table S1: Real and modeled data on cases of HMPX in the assessed geographical regions.
